# TSH Levels as an Independent Risk Factor for NAFLD and Liver Fibrosis in the General Population

**DOI:** 10.3390/jcm10132907

**Published:** 2021-06-29

**Authors:** Alba Martínez-Escudé, Guillem Pera, Anna Costa-Garrido, Lluís Rodríguez, Ingrid Arteaga, Carmen Expósito-Martínez, Pere Torán-Monserrat, Llorenç Caballería

**Affiliations:** 1 Unitat de Suport a la Recerca (USR) Metropolitana Nord, Fundació Institut Universitari d’Investigació en Atenció Primària Jordi Gol i Gurina (IDIAP Jordi Gol), Carrer de la Mare de Déu de Guadalupe 2, Planta 1ª, 08303 Mataró, Spain; gpera@idiapjgol.info (G.P.); annacostaga@gmail.com (A.C.-G.); lrodriguezg@gencat.cat (L.R.); iarteaga@gencat.cat (I.A.); cexposito.mn.ics@gencat.cat (C.E.-M.); ptoran.bnm.ics@gencat.cat (P.T.-M.); lcaballeria.bnm.ics@gencat.cat (L.C.); 2 Centre d’Atenció Primària La Llagosta, Institut Català de la Salut, 08120 La Llagosta, Spain; 3 Centro de Investigación Biomédica en Red de Enfermedades Hepáticas y Digestivas (CIBEREHD), 28029 Madrid, Spain; 4 Facultat de Matemàtiques, Universitat Autònoma de Barcelona, 08290 Cerdanyola del Vallès, Spain; 5 Centre d’Atenció Primària Rocafonda-Palau, Institut Català de la Salut, 08303 Mataró, Spain; 6 Centre d’Atenció Primària Santa Eulàlia, Institut Català de la Salut, 08187 Santa Eulàlia de Ronçana, Spain; 7 Centre d’Atenció Primària Badia del Vallès, Institut Català de la Salut, 08214 Badia del Vallès, Spain

**Keywords:** thyroid stimulating hormone, thyroid function, metabolic syndrome, obesity, liver fibrosis, transient elastography, non-alcoholic fatty liver disease

## Abstract

Thyroid hormones may be a risk factor for the development of non-alcoholic fatty liver disease (NAFLD) and its progression to liver fibrosis. The aim of this study is to investigate the relationship between thyroid stimulating hormone (TSH) levels, NAFLD, and liver fibrosis in the general population. A descriptive cross-sectional study was performed in subjects aged 18–75 years randomly selected from primary care centers between 2012 and 2016. Each subject underwent clinical evaluation, physical examination, blood tests and transient elastography. Descriptive and multivariate logistic regression analyses were used to identify factors associated with NAFLD and fibrosis. We included 2452 subjects (54 ± 12 years; 61% female). Subjects with TSH ≥ 2.5 μIU/mL were significantly associated with obesity, atherogenic dyslipidemia, metabolic syndrome (MetS), hypertransaminasemia and altered cholesterol and triglycerides. The prevalence of NAFLD and liver fibrosis was significantly higher in subjects with TSH ≥ 2.5 (μIU/mL). We found a 1.5 times increased risk of NAFLD, 1.8 and 2.3 times increased risk of liver fibrosis for cut-off points of ≥8.0 kPa and ≥9.2 kPa, respectively, in subjects with TSH ≥ 2.5 μIU/mL compared with TSH < 2.5 μIU/mL (control group), independent of the presence of MetS. These findings remained significant when stratifying TSH, with values ≥ 10 μIU/mL.

## 1. Introduction

Non-alcoholic fatty liver disease (NAFLD) has become a major public health problem in recent decades, being the most common liver disease worldwide, with a prevalence between 25% and 30% of the adult population [1]. The main cause of the increase in its prevalence is the close relationship between NAFLD and different metabolic disorders, such as obesity, type 2 diabetes (T2DM), hypertriglyceridemia or metabolic syndrome (MetS), which in turn affect a large number of subjects today [2]. The heterogenicity in the distribution of the disease according to sub-populations with metabolic risk factors has led to a rethinking, even in the nomenclature of NAFLD, with the aim of identifying those patients with a higher risk of liver progression [3].

The main characteristic of NAFLD is fatty infiltration in more than 5% of hepatocytes, which is known as simple steatosis, in the absence of other chronic liver diseases (viral, alcoholic, drug, autoimmune) [4]. Later, this disease can progress to steatohepatitis, with different degrees of affectation, leading to the development of advanced liver fibrosis in 5–8% of patients [5,6]. Detecting liver fibrosis early is crucial as the severity of fibrosis predicts the development of liver cirrhosis and long-term survival.

Among the multiple extrahepatic complications that have been described in NAFLD, where metabolic and endocrinological disorders predominate, are alterations in thyroid function [7]. Thyroid hormones (TH) are involved in glycemic and lipid metabolism, as well as insulin resistance. The participation of TH in some physiopathological processes of NAFLD, such as the beta oxidation of free fatty acids, the cascade of pro-inflammatory cytokines, oxidative stress reactions or activation of stellate liver cells that lead to a fibrogenic response, make a possible relationship plausible [8]. Some studies have shown that hypothyroidism is more common in subjects with NAFLD [9]; others, that a low thyroid function is related to the risk of developing NAFLD, independently of other metabolic factors [10]. TH levels have also been associated with NAFLD [11] and liver fibrosis [12]. However, due to the heterogenicity of the populations studied and the different diagnostic criteria for defining the alteration of thyroid function and NAFLD or liver fibrosis, other authors have not found such a relationship [13,14]. Therefore, there is still some controversy regarding this association and more studies are required to clarify the role of TH in NAFLD.

For this reason, the main objective of this study is to analyze the relationship between the levels of thyroid stimulating hormone (TSH), NAFLD, and liver fibrosis in the general population.

## 2. Methods

### 2.1. Study Design and Population

For the design of this study, the cohort of a previous project was used, which was carried out between 2012 and 2016, and whose results were recently published [15]. A descriptive, cross-sectional, multi-center, population-based study was carried out, which included subjects aged 18 to 75 years, randomly selected from the Primary Care Information System (SIAP). All subjects were invited to participate in the study by telephone and underwent a clinical interview, a physical examination, a blood test, and a transient liver elastography, with prior informed consent.

To carry out this sub-study, 3060 participants from the reference cohort were selected. Individuals with known chronic liver disease, hepatotoxic drug use, severe advanced disease, cognitive impairment, institutionalization, and death were previously excluded. For this sub-study, only subjects with information on TSH levels were included. The specific exclusion criteria were: incomplete laboratory data (*n* = 202), absence or invalidity of liver elastography measurements (*n* = 46), inability to calculate the Fatty Liver Index (FLI) (*n* = 50) and weekly alcohol consumption of ≥21 standard drink units (SDUs) in men and ≥14 SDUs in women (*n* = 310). Finally, the sample obtained for data analysis was 2452 individuals.

### 2.2. Clinical and Laboratory Parameters

The variables that were collected were the following: age, sex, height, weight, waist circumference (WC), body mass index (BMI), systolic and diastolic blood pressure (SBP and DBP), tobacco and alcohol consumption in SDU. Individuals were also questioned for the presence of previous co-morbidities: arterial hypertension (HBP), hypercholesterolemia, hypertriglyceridemia and T2DM. These data were compared with the records in the computerized medical history. Blood tests were performed after a 12-h fast, including the determination of a complete blood count, TSH, alanine aminotransferase (ALT), aspartate aminotransferase (AST), gamma glutamyltransferase (GGT), alkaline phosphatase (ALP), ferritin, total proteins, albumin, glycemia, glycosylated hemoglobin, total cholesterol, high-density lipoprotein (HDL), low-density lipoprotein (LDL), and triglycerides (TG). Hypertransaminasemia was defined with ALT and/or AST values > 35 U/L, using the cut-off point of our reference laboratory. Atherogenic dyslipidemia was established when TG levels ≥ 150 mg/dL and HDL levels < 40/50 mg/dL in men/women, respectively [16,17]. The normal range used for TSH values was 0.35–4.94 μIU/mL according to data from our reference laboratory.

### 2.3. Evaluation of MetS

The diagnosis of MetS was made according to the criteria established by the NCEP-ATPIII [18] when the subjects presented ≥ 3 of the following components: WC > 88 cm women and > 102 cm men; TG ≥ 150 mg/dL or on lipid-lowering treatment; HDL < 40 mg/dL in men and < 50 mg/dL in women or on lipid-lowering treatment; BP ≥ 130/85 mmHg or on hypotensive treatment; and baseline glycemia ≥ 100 mg/dL or on hypoglycemic treatment.

### 2.4. Evaluation of NAFLD

NAFLD was diagnosed using the FLI serological marker that includes the variables TG, BMI, GGT and WC; and is calculated from the following formula: FLI = (e^0.953 × loge(TG) + 0.139 × BMI + 0.718 × loge(GGT) + 0.053 × WC − 15.745^)/(1 + e^0.953 × loge(TG) + 0.139 × BMI + 0.718 × loge(GGT) + 0.053 × WC − 15.745^) × 100. When the FLI score ≥ 60 the diagnosis is NAFLD, FLI between 30 and 60 the diagnosis is indeterminate, and if FLI < 30 no NAFLD [19].

### 2.5. Evaluation of Liver Fibrosis

Transient liver elastography (TE) was performed on each subject, using the M probe of the Fibroscan 402 apparatus (Echosens, Paris, France). Exclusion criteria were the inability to obtain 10 valid measurements and/or an interquartile range/liver stiffness (LS) measurement ratio greater than 30%. Two cut-off points, suggestive of significant liver fibrosis, were defined according to LS values: ≥8.0 kilopascals (kPa) and ≥9.2 kPa [15,20].

### 2.6. Statistical Analysis

For the descriptive analysis, the continuous variables were expressed as means and standard deviation, since they followed a normal distribution, and the categorical variables in frequencies and percentages. The prevalences were calculated with their respective 95% confidence intervals (95% CI). In the bivariate comparisons of categorical variables, the Chi-square test was used, for the continuous variables in two groups the Student’s *t*-test was used, and for comparisons in four groups analysis of variance was used.

The main outcome variables were, on the one hand, the presence of NAFLD (FLI ≥ 60), and on the other, liver fibrosis, for which the LS measured by TE was used at two cut-off points (≥8.0 kPa and ≥9.2 kPa) used independently for the analyses. Subjects were stratified into various groups, according to TSH levels, to assess the risk of presenting NAFLD or liver fibrosis. The main TSH groups (model 1) for the study of the outcome variables were: TSH < 2.5 μIU/mL, corresponding to the control group, and TSH ≥ 2.5 μIU/mL, as an explanatory variable. Furthermore, in order to better define the subjects with TSH ≥ 2.5 μIU/mL, they were additionally sub-classified into three groups (model 2): TSH 2.50–4.94 μIU/mL, TSH 4.95–9.99 μIU/mL and TSH ≥ 10 μIU/mL. Multivariate logistic regression analyses were used in several models adjusted for potential confounding factors. The corresponding odds ratio (OR) and their 95% CI were obtained. Statistical tests were performed with bilateral contrasts and statistical significance of *p* < 0.05. The analyses were carried out with the R package version 4.0.2 (R development Core Team, GNU, GPL) and Rstudio version 1.2.5019 (R Foundation for Statistical Computing, Vienna, Austria).

## 3. Results

### 3.1. Basal Characteristics

Of the 2452 subjects included in this study, 61% were female, 94% Caucasian, and they had a mean age of 54 ± 12 years. The prevalence of the different metabolic factors in the global sample were the following: T2DM 10%, HBP 26%, hypercholesterolemia 38%, obesity 31%, hypertriglyceridemia 11% and abdominal obesity 47%. In addition, 27% of the subjects presented diagnostic criteria for MetS. Hypertransaminasemia affected 13% of the sample and NAFLD was found in 35% of the cases.

The individuals were classified according to TSH levels into two groups (model 1): 66% with TSH < 2.5 μIU/mL (*n* = 1619) and 44% with TSH ≥ 2.5 μIU/mL (*n* = 833). Additionally, subjects with TSH ≥ 2.5 μIU/mL were stratified in (model 2): 86% with TSH 2.50–4.94 μIU/mL (*n* = 718); 12% with TSH 4.95–9.99 μIU/mL (*n* = 96) and 2% with TSH ≥ 10 μIU/mL (*n* = 19).

The baseline characteristics of the study subjects are shown in Table 1. Obesity, both global and abdominal, hypercholesterolemia, atherogenic dyslipidemia, MetS, and hypertransaminasemia were significantly more prevalent in the group with TSH ≥ 2.5 μIU/mL compared to the control group. Likewise, in these subjects higher levels of BMI, WC, total cholesterol, TG, ALT, AST, and ALP were observed. Similar findings were observed in the stratification of the subjects with TSH ≥ 2.5 μIU/mL in model 2. Furthermore, in the group with TSH ≥ 10 μIU/mL, a higher prevalence of T2DM was found compared to the control group (16% vs. 9.8%) and higher LDL levels (*p* < 0.001). However, although the prevalence of MetS in subjects with TSH ≥ 10 μIU/mL was higher (32% vs. 25%), it was not significant.

### 3.2. Relationship between TSH and NAFLD

First, the relationship of TSH levels according to the presence of NAFLD was studied (Table 2). TSH levels were significantly higher in subjects with FLI ≥ 60 (2.9 vs. 2.3 μIU/mL). The prevalence of subjects with TSH ≥ 2.5 μIU/mL was higher in the presence of NAFLD compared to the group without NAFLD (39% vs. 31%; *p* < 0.001). The prevalence of TSH alteration, in the stratified groups of model 2, was also higher in the group with NAFLD (*p* < 0.001).

**Figure 1:**
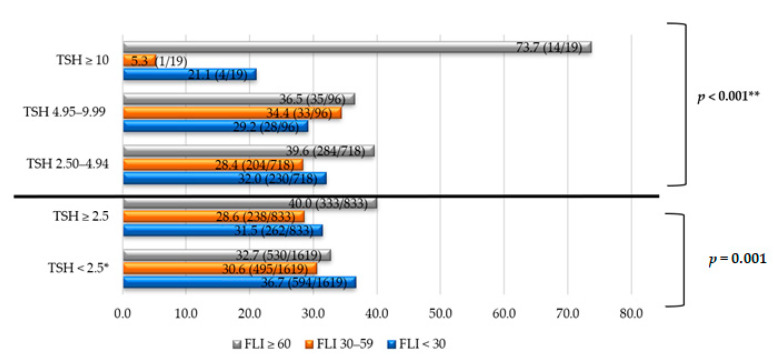
NAFLD prevalence according to TSH levels in different groups. Note: The data are expressed in %. * Comparison/control group. ** *p* trend, comparing four groups of TSH: < 2.5, 2.50–4.94, 4.95–9.99, ≥ 10 μIU/mL. TSH units: μIU/mL. Abbreviations: NAFLD, non-alcoholic fatty liver disease; TSH, thyroid stimulating hormone; FLI, fatty liver index.

Furthermore, the prevalence of NAFLD was analyzed according to TSH levels (Figure 1). NAFLD affected 40% of the subjects with TSH ≥ 2.5 μIU/mL (*p* = 0.001). Moreover, NAFLD occurred more frequently when TSH levels were higher (73.7% in subjects with TSH ≥ 10 μIU/mL).

**Figure 2:**
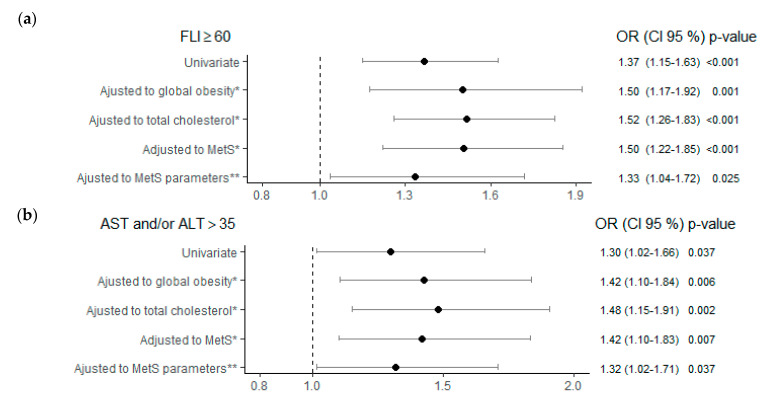
Risk of NAFLD (**a**) and hypertransaminasemia (**b**) according to TSH ≥ 2.5 (μIU/mL) adjusted to different metabolic factors. Note: Control reference group for the analysis TSH < 2.5 (μIU/mL). * All multivariate analyses were adjusted also for age, sex and alcohol consumption. ** MetS parameters: WC > 88W/102M cm; TG ≥ 150 mg/dL; HDL < 50 W/40 M mg/dL; BP ≥ 130/85 mmHg; Glyc ≥ 100 mg/dL. Abbreviations: FLI, fatty liver index; MetS, metabolic syndrome; AST, aspartate aminotransferase; ALT, alanine aminotransferase; TSH, thyroid stimulating hormone; NAFLD, non-alcoholic fatty liver disease; WC, waist circumference; TG, triglycerides; HDL, high-density lipoprotein; BP, blood pressure; Glyc, glycemia.

In multivariate analyses, the risk of presenting NAFLD according to TSH levels ≥ 2.5 μIU/mL, compared to the control group, was 1.5 times greater regardless of age, sex, alcohol consumption, obesity, cholesterol, or MetS; and 1.33 times greater, regardless of the MetS parameters (Figure 2a).

To better explain these findings, the risk of NAFLD was analyzed in the different stratified TSH groups of model 2. Both the subjects with TSH 2.50–4.94 μIU/mL and those with TSH ≥ 10 μIU/mL showed a significant increase in the risk of NAFLD of 1.31 and 8.22 times, respectively, in relation to the control group, independently of age, sex, alcohol consumption, and the different parameters of MetS (Table 3). In addition, an excess risk of FLI ≥ 60 with OR > 1 was also obtained in these two TSH groups, in the other multivariate analyses carried out in separate models and adjusted for global obesity, total cholesterol, and presence of MetS. Although the ORs for the TSH 4.95–9.99 μIU/mL group were >1, they did not reach statistical significance.

### 3.3. Relationship between TSH and Hypertransaminasemia

The prevalence of hypertransaminasemia (ALT and/or AST > 35 U/L) increased from 12%, in subjects with TSH < 2.5 μIU/mL, to 15% in subjects with TSH ≥ 2.5 μIU/mL or 26% when TSH ≥ 10 μIU/mL, in a significant way (*p* = 0.036 and *p* = 0.040, respectively). ALT and ALP values underwent a dose-dependent increase in relation to TSH levels (Table 1).

The risk of presenting hypertransaminasemia when TSH ≥ 2.5 μIU/mL was higher compared to the control group, and independent of age, sex, obesity, cholesterol, MetS, or MetS parameters (Figure 2b). In the multivariate analyses of model 2, the subjects with TSH 2.50–4.94 μIU/mL presented an increased risk of hypertransaminasemia of 1.34 times, compared to the control group, independently of the different parameters of MetS (*p* = 0.035). Furthermore, the risk of ALT and/or AST > 35 U/L in subjects with TSH ≥ 10 μIU/mL was 2.24 times higher compared to the TSH < 2.5 μIU/mL group, although these results were not significant (Table 4).

### 3.4. Association between TSH and Liver Fibrosis

LS values increased with TSH levels: from 4.8 ± 1.7 kPa in the group with TSH < 2.5 μIU/mL to 5.1 ± 2.7 kPa in subjects with TSH ≥ 2.5 μIU/mL (*p* = 0.003). A significant increase in LS was also observed in the group with TSH ≥ 10 μIU/mL (LS 5.3 ± 1.8 kPa, *p* = 0.017).

**Figure 3:**
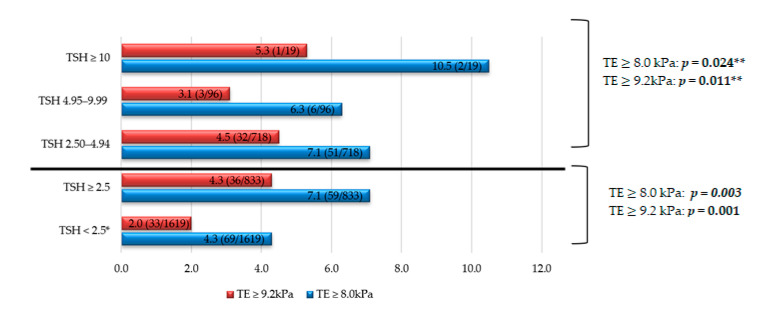
Liver fibrosis prevalence according to TSH levels in different groups. Note: The data are expressed in %. * Comparison/control group. ** *p* trend, comparing four groups of TSH: <2.5, 2.50–4.94, 4.95–9.99, ≥10 μIU/mL. TSH units: μIU/mL. Abbreviations: TE, transient elastography; TSH, thyroid stimulating hormone.

Regarding the prevalence of fibrosis, as seen in Figure 3, higher values are reached in all TSH groups compared to the control group. Some 7.1% and 4.3% of the subjects with TSH ≥ 2.5 μIU/mL obtained TE values ≥ 8.0 kPa and ≥9.2 kPa, respectively, higher than the control group. Furthermore, in subjects with TSH ≥ 10 μIU/mL, the prevalence of fibrosis was higher: Some 10.5% vs. 4.3% (control group) for TE values ≥ 8.0 kPa; and 5.3% vs. 2.0% (control group) for TE values ≥ 9.2 kPa. All of these findings were statistically significant.

**Figure 4:**
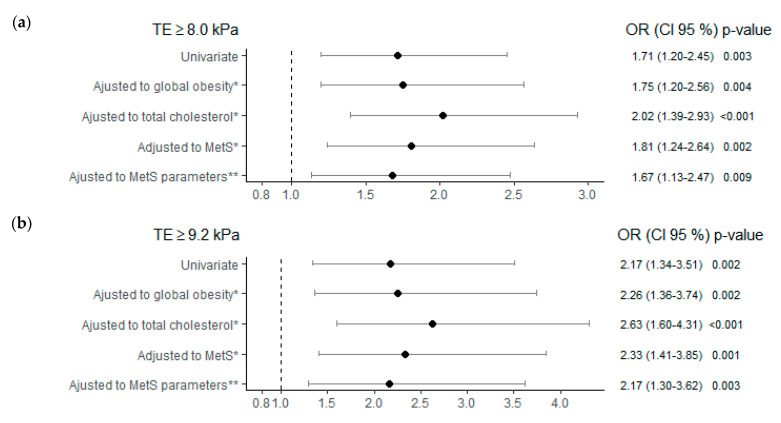
Liver fibrosis risk, using two transient elastography cut-offs, TE ≥ 8.0kPa in (**a**) and TE ≥ 9.2 kPa in (**b**), as dependent variables, in TSH group ≥ 2.5 (μIU/mL), adjusted to different metabolic factors. Note: Control reference group for the analysis TSH < 2.5 (μIU/mL). * All multivariate analyses were adjusted also for age, sex and alcohol consumption. ** MetS parameters: WC > 88 W/102 M cm; TG ≥ 150 mg/dL; HDL < 50 W/40 M mg/dL; BP ≥ 130/85 mmHg; Glyc ≥ 100 mg/dL. Abbreviations: MetS, metabolic syndrome; TE, transient elastography; TSH, thyroid stimulating hormone; WC, waist circumference; TG, triglycerides; HDL, high-density lipoprotein; BP, blood pressure; Glyc, glycemia.

The risk of fibrosis, at both TE cut-off points, in subjects with TSH ≥ 2.5 μIU/mL was higher when compared to the group with TSH < 2.5 μIU/mL, regardless of age, sex, obesity, cholesterol, and MetS (Figure 4). Subjects with TSH ≥ 2.5 μIU/mL showed an increased risk of fibrosis of 1.67 (Figure 4a) and 2.17 times (Figure 4b) for the cut-off points TE ≥ 8.0 kPa and ≥ 9.2 kPa respectively and independent of the different parameters of MetS.

Likewise, in the multivariate analyses carried out with the different TSH groups (model 2), an increased risk of fibrosis was determined, both in TE ≥ 8.0 kPa and ≥ 9.2 kPa, in subjects with TSH 2.50–4.94 μIU/mL, significantly and independently of all the confounding factors studied (Table 5). Although in the TSH groups with higher levels an increased risk of fibrosis was also observed for both cut-off points, these findings were not significant for the TSH 4.95–9.99 μIU/mL groups or for TSH ≥ 10 μIU/mL, with the exception of the analysis adjusted for total cholesterol.

## 4. Discussion

The findings of this study demonstrate the association between TSH levels, NAFLD, and liver fibrosis. Specifically, those subjects with TSH levels ≥ 2.5 μIU/mL have a significantly increased risk of presenting NAFLD and fibrosis independently of the different metabolic factors studied.

The relationship between thyroid function or TH and NAFLD was analyzed in several studies in the last decade. There are multiple differences in the methodology used to study both pathologies. Thus, there are studies that have used serological markers, such as FLI, for the diagnosis of NAFLD [21], while others have used abdominal ultrasound [13] or pathological criteria [22]. The most accepted upper limit of TSH to define normality of thyroid function is between 4 and 5 μIU/mL [23], although there are authors who differentiate subjects with an upper limit of normality of TSH < 2.5 μIU/mL as those with strictly normal thyroid function [24]. Other factors, such as race, the presence of antithyroid antibodies, or the percentage of patients undergoing hormone replacement therapy, also influence the variability of the studies carried out.

Some studies have shown higher TSH levels [10] or a higher prevalence of hypothyroidism [25] in subjects with NAFLD. A study was recently published, with a large sample of subjects, which shows a higher incidence of hypothyroidism and autoimmune thyroiditis in the group with NAFLD, with a follow-up period of 10 years [26]. Other studies confirm an increased risk of presenting NAFLD based on TSH levels [27] or thyroid function [28]. Low T4 [29] or high T3 [30] levels have been correlated with liver steatosis. Furthermore, TSH levels have also been linked to NASH [31]. Likewise, there are studies that have demonstrated these associations independently of MetS [32] and, on the other hand, others that have not [33].

Furthermore, the relationship between TH and NAFLD has also been studied in specific subpopulations according to some metabolic conditioning factors. In the morbidly obese population, an increased risk of NAFLD, defined by serological markers, has been found in subjects with higher TSH and T3 values [34]. Higher TSH levels, lower T4 levels and a higher prevalence of positivity of anti-thyroid peroxidase antibodies were found in patients where T2DM and NAFLD coexist, compared to those without NAFLD [35]. In another similar study, carried out in a diabetic and euthyroid population, it was shown that subjects with higher levels of T3 and TSH had a higher risk of presenting NAFLD, with ORs of 3.02 and 1.58, respectively [36].

The meta-analyses available to date show some different results. In one by Guo et al., where 26 studies were included, the subjects with NAFLD/NASH reached significantly higher levels of TSH in relation to the control group. In addition, a 1.6 times increased risk of NAFLD was also demonstrated in hypothyroid subjects [37]. Along the same lines, Mantovani et al. demonstrated the association between hypothyroidism and NAFLD independently of age, sex, BMI, and other metabolic factors studied, in a study involving 44,140 individuals [38]. He et al. also found an increased risk of NAFLD of 1.81 and 1.63 times in subjects with overt and subclinical hypothyroidism, respectively [39]. In contrast, the study by Jaruvongvanich et al. did not demonstrate a relationship between thyroid hormones (TSH, T4, T3) and NAFLD, nor with hypothyroidism [40]. The findings of our study are in line with the above. On the one hand, we found a correlation between NAFLD and TSH levels; on the other hand, a higher prevalence of NAFLD in subjects with higher TSH and lastly, an increased risk of NAFLD of 1.5 times in subjects with TSH ≥ 2.5 μIU/mL independently of MetS. This last finding is of interest because it shows a higher risk of NAFLD in patients with TSH levels that are within normal parameters.

Although there is still some controversy in the relationship between TH and NAFLD, it is plausible to think that both entities are linked, either by a direct effect of TH or by an effect mediated by the components of MetS. A low thyroid function is associated with increases in cholesterol and TG levels, and with greater weight gain, which in turn are risk factors for the development of NAFLD [41]. We have demonstrated these same findings in our study. At the pathophysiological level, THs participate in intrahepatic lipolysis, through the activation of autophagy, and the beta oxidation of fatty acids [42], but when there is a low thyroid function, the activity of hepatic lipases decreases which entails accumulation of TG in hepatocytes [43]. Furthermore, it has been shown that, in hypothyroid subjects, adipocytokine levels are altered [44], which can contribute to liver inflammation processes. THs have also been linked to the regulation of micro-RNAs [45] and to some genetic polymorphisms related to NAFLD. For example, a recent study has shown that there is a significant association between euthyroid subjects with high-normal TSH (2.5 to 5.3 μIU/mL) and NASH, when they carry the risk of allele of PNPLA3 G [46].

THs have been correlated with moderate elevations in transaminase levels. In an observational study in our setting, where 10,116 subjects were included, it was found that the alteration in AST levels affected subjects with TSH ≥ 10 µIU/mL more frequently and significantly; it was also more prevalent in subjects with low T4 levels [41]. Along the same lines, Chung et al. demonstrated the association between alteration of ALT > 33/25 (men/women) and thyroid function, affecting 20.1% of subjects with subclinical hypothyroidism and 25.9% in clinical hypothyroidism [10]. Furthermore, ALT levels have also been linked to MetS [47]. Similarly, our study found a 1.32 times increased risk of having hypertransaminasemia in subjects with TSH ≥ 2.5 μIU/mL, regardless of MetS parameters. Although hypertransaminasemia is not a good predictive marker of NAFLD, since there may be steatosis with normal liver function, we believe that the relationship found between TH and hypertransaminasemia is due to the association with NAFLD.

Moreover, the role of TH in liver fibrosis has also been discussed. At the pathophysiological level, it is speculated that TH may be involved in the activation of stellate liver cells, a crucial step in the development of liver fibrosis [48]. In the same way as the studies that relate thyroid function and NAFLD, in this case, the methods used for the diagnosis of liver fibrosis are also different. While some authors use serological markers [34,49], others use TE [50] or liver biopsy [51]. In our case, we used TE, which has a high sensitivity and specificity and is the usual screening method in our setting. The findings that we encountered are similar to those present in the literature. Bano et al. found that higher TSH levels were associated with a 1.49 times increased risk of liver fibrosis, using TE values ≥ 8.0 kPa as the cut-off point [50]. Kim et al. also found a relationship between low thyroid function and liver fibrosis, using serological markers [49]. In another recent study, low-normal thyroid function was associated with TE values ≥ 8.0 kPa and ≥9.2 kPa, in euthyroid subjects, dependent on other metabolic factors [52]. Along the same lines, in a recently published meta-analysis, hypothyroidism, both sub-clinical and overt, was associated with an increased risk of liver fibrosis with OR > 2 [53]. In our study, we found an independent relationship of MetS parameters between subjects with TSH ≥ 2.5 μIU/mL and liver fibrosis. Still, these findings need to be confirmed in prospective studies.

Based on this evidence, in recent years it has been proposed to consider TH or the thyroid hormone receptor (THR) as therapeutic targets for NAFLD. In a population study with dyslipidemia and sub-clinical hypothyroidism, the use of low doses of levothyroxine decreased the prevalence of NAFLD [54]. In another, a 12% reduction in intrahepatic lipids was demonstrated in subjects with NAFLD and T2DM when using levothyroxine [55]. Treatments with agonists of THR-β, which is the main receptor for TH in the liver, have also been tried to reduce cholesterol levels [56] or for the treatment of non-alcoholic steatohepatitis [57]. Even so, no treatment has yet been approved for NAFLD and non-pharmacological measures aimed at controlling metabolic factors continue to be the gold standard in the treatment of these patients.

Finally, this study has some limitations. The gold standard method for the diagnosis of NAFLD and liver fibrosis is liver biopsy, but as it is an invasive test, it is not performed in routine clinical practice [4]. In this specific case, validated serological markers have been used for the diagnosis of NAFLD and liver stiffness measurements using transient liver elastography for the diagnosis of fibrosis. Furthermore, the recommended probe to measure liver stiffness in obese subjects is the XL, but in our study all the examinations were performed with the M probe as it was the only probe available. Due to the design of the reference cohort used, we do not know the percentage of subjects who were being treated with hormone replacement therapy, and we do not have information on T4 or T3 levels, the presence of antithyroid antibodies or the previous diagnosis of thyroid cancer. Finally, the cross-sectional design of this study does not allow for determining causal relationships.

## 5. Conclusions

In conclusion, the findings of this study demonstrate that TSH levels ≥ 2.5 μIU/mL are associated with a higher risk of NAFLD and liver fibrosis in the general population, independently of the MetS parameters. Furthermore, individuals with TSH ≥ 10 μIU/mL have an additional increased risk of NAFLD. Although more prospective design studies are required, we propose the need for stricter control of TSH levels in those subjects with coexisting metabolic risk factors for developing NAFLD and liver fibrosis.

## Figures and Tables

**Table 1:** Basal characteristics of the subjects according to TSH levels (μIU/mL).

	TSH < 2.5 (*n* = 1619)	TSH ≥ 2.5 (*n* = 833)	*p*-Value	TSH ≥ 2.5			
				TSH 2.50–4.94 (*n* = 718)	TSH 4.95–9.99 (*n* = 96)	TSH ≥ 10 (*n* = 19)	*p*-Value *
Age (years)	54 ± 12	55 ± 12	0.013	55 ± 12	56 ± 11	52 ± 9	0.042
Female	909 (56%)	592 (71%)	<0.001	503 (70%)	76 (79%)	13 (68%)	<0.001
Disease history							
T2DM	158 (9.8%)	95 (11%)	0.205	88 (12%)	4 (4.2%)	3 (16%)	0.046
HBP	421 (26%)	221 (27%)	0.779	201 (28%)	15 (16%)	5 (26%)	0.079
Hypercholesterolemia	599 (37%)	344 (41%)	0.038	299 (42%)	36 (38%)	9 (47%)	0.157
Hypertriglyceridemia	163 (10%)	101 (12%)	0.120	88 (12%)	12 (13%)	1 (5.3%)	0.337
Atherogenic dyslipidemia	139 (9.0%)	107 (13%)	0.001	90 (13%)	14 (15%)	3 (16%)	0.009
Global obesity (BMI ≥ 30)	454 (28%)	294 (35%)	<0.001	250 (35%)	34 (35%)	10 (53%)	0.007
Abdominal obesity	705 (44%)	436 (52%)	<0.001	370 (52%)	53 (55%)	13 (68%)	<0.001
MetS	405 (25%)	249 (30%)	0.010	216 (30%)	27 (28%)	6 (32%)	0.076
Physical examination							
BMI	28 ± 5	29 ± 5	<0.001	29 ± 5	29 ± 5	30 ± 6	0.001
WC-Male (cm)	98 ± 11	100 ± 12	0.032	100 ± 12	100 ± 13	101 ± 8	0.201
WC-Female (cm)	90 ± 12	92 ± 13	0.001	91 ± 13	93 ± 12	96 ± 15	0.003
SBP (mmHg)	125 ± 17	125 ± 17	0.801	125 ± 17	126 ± 16	126 ± 19	0.903
DBP (mmHg)	80 ± 10	81 ± 10	0.013	81 ± 10	81 ± 11	83 ± 13	0.066
Blood analysis							
Platelets (10^9^/L)	244 ± 59	246 ± 60	0.471	245 ± 61	253 ± 60	251 ± 47	0.527
ALT (U/L)	23 ± 13	24 ± 16	0.047	24 ± 16	22 ± 14	29 ± 19	0.040
AST (U/L)	23 ± 8	24 ± 10	0.025	24 ± 10	23 ± 8	25 ± 8	0.058
ALT and/or AST > 35 (U/L)	189 (12%)	122 (15%)	0.036	107 (15%)	10 (10%)	5 (26%)	0.040
GGT (U/L)	30 ± 26	31 ± 32	0.172	32 ± 32	27 ± 25	36 ± 40	0.233
ALP (U/L)	77 ± 24	79 ± 25	0.019	79 ± 25	83 ± 24	89 ± 15	0.008
Ferritin (ng/mL)	115 ± 114	105 ± 110	0.045	106 ± 110	104 ± 114	82 ± 63	0.181
Glycemia (mg/dL)	100 ± 24	101 ± 28	0.181	101 ± 28	100 ± 32	96 ± 21	0.418
HbA1c (%)	5.7 ± 0.7	5.7 ± 0.7	0.199	5.7 ± 0.7	5.7 ± 0.8	5.6 ± 0.3	0.479
Total cholesterol (mg/dL)	210 ± 39	216 ± 38	<0.001	214 ± 39	225 ± 35	243 ± 37	<0.001
HDL (mg/dL)	55 ± 13	56 ± 13	0.018	56 ± 13	56 ± 12	53 ± 7	0.069
LDL (mg/dL)	133 ± 34	135 ± 34	0.176	133 ± 33	144 ± 32	161 ± 39	<0.001
TG (mg/dL)	115 ± 68	130 ± 78	<0.001	129 ± 77	133 ± 82	154 ± 58	<0.001

*p comparing four groups of TSH: < 2.5, 2.50–4.94, 4.95–9.99 and ≥ 10 μIU/mL. Note: The data are expressed in frequency (%) or mean (SD). MetS diagnosed by NCEP-ATPIII criteria. Abbreviations: TSH, thyroid stimulating hormone; T2DM, type 2-diabetes mellitus; HBP, high blood pressure; MetS, metabolic syndrome; BMI, body mass index; WC, waist circumference; SBP, systolic blood pressure; DBP, diastolic blood pressure; ALT, alanine aminotransferase; AST, aspartate aminotransferase; GGT, g-glutamyltransferase; ALP, alkaline phosphatase; HbA1c, glycosylated hemoglobin; HDL, high density lipoprotein; LDL, low-density lipoprotein; TG, triglycerides.

**Table 2:** TSH levels and prevalence of TSH alteration according to presence of NAFLD.

	FLI < 60 (*n* = 1589)	FLI ≥ 60 (*n* = 863)	*p*-Value
TSH level (μIU/mL)	2.3 ± 2.4	2.9 ± 6.2	<0.001
Groups TSH (*n*, %)			
TSH < 2.5 (μIU/mL)	1089 (69%)	530 (61%)	<0.001
TSH ≥ 2.5 (μIU/mL)	500 (31%)	333 (39%)	
TSH 2.50–4.94 (μIU/mL)	434 (27%)	284 (33%)	<0.001 *
TSH 4.95–9.99 (μIU/mL)	61 (3.8%)	35 (4.1%)	
TSH ≥ 10 (μIU/mL)	5 (0.3%)	14 (1.6%)	

* *p* trend, comparing four groups of TSH: <2.5, 2.50–4.94, 4.95–9.99, ≥10 μIU/mL. Abbreviations: FLI, fatty liver index; TSH, thyroid stimulating hormone.

**Table 3:** Analysis of NAFLD risk according to TSH group. Four different multivariate logistic regression models, adjusting for obesity, cholesterol, MetS and MetS parameters.

	FLI ≥ 60
	OR (CI 95%) *p*-value
Univariate	
TSH 2.50–4.94 (μIU/mL)	1.34 (1.12–1.61) 0.001
TSH 4.95–9.99 (μIU/mL)	1.18 (0.77–1.81) 0.451
TSH ≥ 10 (μIU/mL)	5.75 (2.06–16.06) < 0.001
Multivariate *	
*Adjusted to global obesity*	
TSH 2.50–4.94 (μIU/mL)	1.46 (1.13–1.89) 0.004
TSH 4.95–9.99 (μIU/mL)	1.28 (0.71–2.31) 0.410
TSH ≥ 10 (μIU/mL)	8.71 (2.51–30.25) < 0.001
*Adjusted to total cholesterol*	
TSH 2.50–4.94 (μIU/mL)	1.49 (1.23–1.81) < 0.001
TSH 4.95–9.99 (μIU/mL)	1.29 (0.83–2.02) 0.262
TSH ≥ 10 (μIU/mL)	6.75 (2.38–19.13) < 0.001
*Adjusted to MetS*	
TSH 2.50–4.94 (μIU/mL)	1.45 (1.16–1.80) < 0.001
TSH 4.95–9.99 (μIU/mL)	1.34 (0.82–2.19) 0.247
TSH ≥ 10 (μIU/mL)	9.33 (3.11–27.97) < 0.001
Adjusted to MetS parameters **	
TSH 2.50–4.94 (μIU/mL)	1.31 (1.00–1.71) 0.046
TSH 4.95–9.99 (μIU/mL)	1.14 (0.63–2.06) 0.655
TSH ≥ 10 (μIU/mL)	8.22 (1.77–38.04) 0.007

Note: Control reference group for the analysis TSH < 2.5 (μIU/mL). * All multivariate analyses were adjusted also for age, sex and alcohol consumption. ** MetS parameters: WC > 88 W/102 M cm; TG ≥ 150 mg/dL; HDL < 50 W/40 M mg/dL; BP ≥ 130/85 mmHg; Glyc ≥ 100 mg/dL. Abbreviations: MetS, metabolic syndrome; FLI, fatty liver index; TSH, thyroid stimulating hormone; WC, waist circumference; TG, triglycerides; HDL, high-density lipoprotein; BP, blood pressure; Glyc, glycemia.

**Table 4:** Analysis between TSH group and risk of hypertransaminasemia.

	ALT and/or AST > 35 U/L
	OR (CI 95%) *p*-value
Univariate	
TSH 2.50–4.94 (μIU/mL)	1.33 (1.03–1.71) 0.031
TSH 4.95–9.99 (μIU/mL)	0.88 (0.45–1.72) 0.709
TSH ≥ 10 (μIU/mL)	2.70 (0.96–7.59) 0.059
Multivariate *	
TSH 2.50–4.94 (μIU/mL)	1.34 (1.02–1.75) 0.035
TSH 4.95–9.99 (μIU/mL)	0.98 (0.49–1.97) 0.962
TSH ≥ 10 (μIU/mL)	2.24 (0.76–6.61) 0.145

Note: Control reference group for the analysis TSH < 2.5 (μIU/mL). * Adjusting for age, sex, alcohol consumption and different parameters of MetS (WC > 88 W/102 M cm; TG ≥ 150 mg/dL; HDL < 50 W/40 M mg/dL; BP ≥ 130/85 mmHg; Glyc ≥ 100 mg/dL). Abbreviations: ALT, alanine aminotransferase; AST, aspartate aminotransferase; TSH, thyroid stimulating hormone; MetS, metabolic syndrome; WC, waist circumference; TG, triglycerides; HDL, high-density lipoprotein; BP, blood pressure; Glyc, glycemia.

**Table 5:** Analysis of liver fibrosis risk, using two elastography cut-offs as dependent variables, according to TSH group. Four different multivariate logistic regression models, adjusting for obesity, cholesterol, MetS and MetS parameters.

	TE ≥ 8.0 kPa	TE ≥ 9.2 kPa
	OR (CI 95%) *p*–value	OR (CI 95%) *p*–value
Univariate		
TSH 2.50–4.94 (μIU/mL)	1.72 (1.18–2.49) 0.004	2.24 (1.37–3.68) 0.001
TSH 4.95–9.99 (μIU/mL)	1.50 (0.63–3.54) 0.358	1.55 (0.47–5.15) 0.474
TSH ≥ 10 (μIU/mL)	2.64 (0.60–11.67) 0.200	2.67 (0.35–20.6) 0.346
Multivariate *		
*Adjusted to global obesity*		
TSH 2.50–4.94 (μIU/mL)	1.74 (1.17–2.58) 0.006	2.30 (1.37–3.87) 0.002
TSH 4.95–9.99 (μIU/mL)	1.72 (0.69–4.25) 0.243	1.82 (0.52–6.36) 0.346
TSH ≥ 10 (μIU/mL)	2.39 (0.50–11.37) 0.272	2.44 (0.29–20.27) 0.410
*Adjusted to total cholesterol*		
TSH 2.50–4.94 (μIU/mL)	1.98 (1.34–2.90) < 0.001	2.64 (1.59–4.39) < 0.001
TSH 4.95–9.99 (μIU/mL)	2.00 (0.83–4.86) 0.124	2.15 (0.63–7.32) 0.222
TSH ≥ 10 (μIU/mL)	5.01 (1.10–22.85) 0.037	5.44 (0.68–43.54) 0.111
*Adjusted to MetS*		
TSH 2.50–4.94 (μIU/mL)	1.76 (1.19–2.61) 0.005	2.32 (1.39–3.88) 0.001
TSH 4.95–9.99 (μIU/mL)	1.94 (0.79–4.75) 0.147	2.17 (0.63–7.51) 0.220
TSH ≥ 10 (μIU/mL)	3.34 (0.71–15.78) 0.128	3.54 (0.43–29.37) 0.241
Adjusted to MetS parameters **		
TSH 2.50–4.94 (μIU/mL)	1.64 (1.10–2.46) 0.016	2.17 (1.28–3.68) 0.004
TSH 4.95–9.99 (μIU/mL)	1.78 (0.72–4.44) 0.213	2.00 (0.57–7.02) 0.277
TSH ≥ 10 (μIU/mL)	2.46 (0.50–12.09) 0.269	2.66 (0.31–22.98) 0.375

Note: Control reference group for the analysis TSH < 2.5 (μIU/mL). * All multivariate analyses were adjusted also for age, sex and alcohol consumption. ** MetS parameters: WC > 88 W/102 M cm; TG ≥ 150 mg/dL; HDL < 50 W/40 M mg/dL; BP ≥ 130/85 mmHg; Glyc ≥ 100 mg/dL. Abbreviations: MetS, metabolic syndrome; TE, transient elastography; TSH, thyroid stimulating hormone; WC, waist circumference; TG, triglycerides; HDL, high-density lipoprotein; BP, blood pressure; Glyc, glycemia.

## Data Availability

Data sharing not applicable.
